# Supplementary figures and images for: Protective role of RIPK1 scaffolding against HDV-induced hepatocyte cell death and the significance of cytokines in mice
Source: PLoS Pathog. 2024 May 13;20(5):e1011749. doi: 10.1371/journal.ppat.1011749 (PMC11115361; doi:10.1371/journal.ppat.1011749)

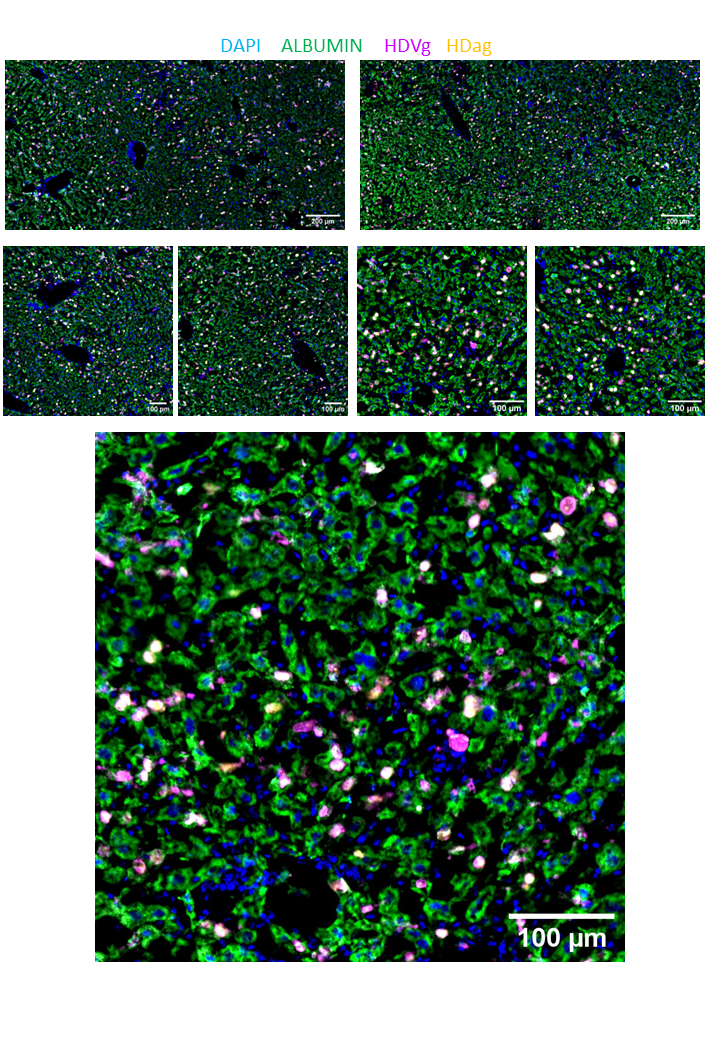

Supplement: S1 Fig — Albumin (green) and HDV RNA genome (HDVg in pink) and antigenome (HDVag in yellow) distribution was analyzed by in situ hybridization (ISH) in the liver of C57BL/6 mice 21 days after receiving adenoassociated viral (AAV) vectors delivering both HBV and HDV genomes (HBV/HDV) at a dose of 5x10^10^ vg/mouse each. Representative images of hybridized liver sections were captured using the Vectra Polaris Automated Imaging System at various magnifications. (TIF) [file ppat.1011749.s001.TIF]

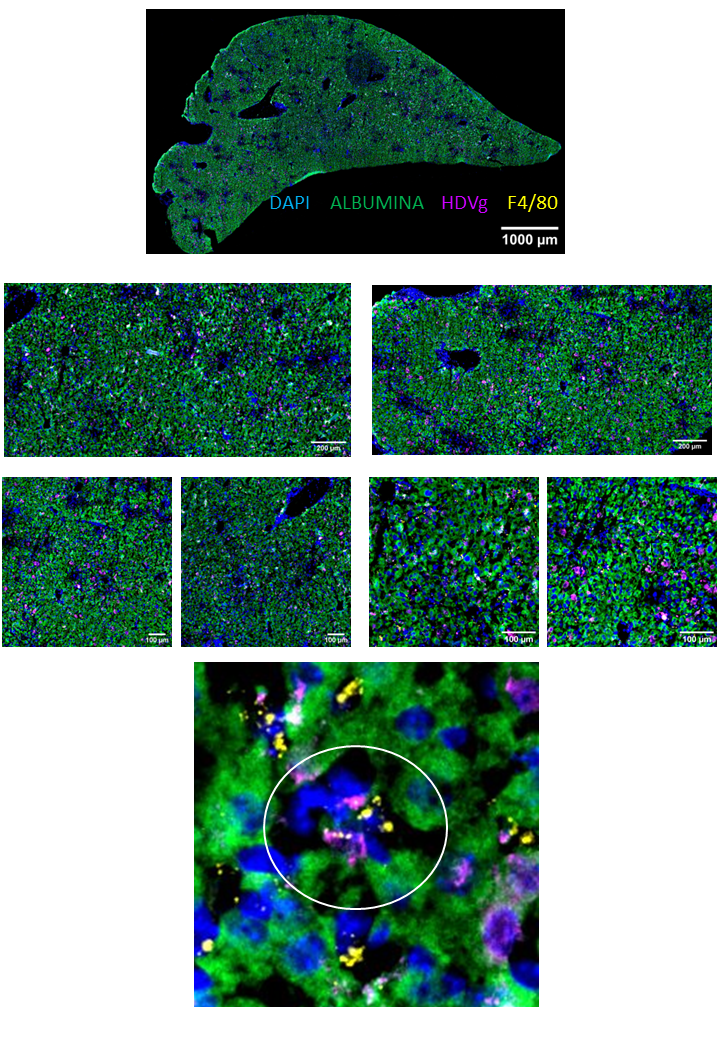

Supplement: S2 Fig — Albumin (green) and HDVg (pink) and F4/80 (yellow) distribution was analyzed by in situ hybridization (ISH) in the liver of C57BL/6 mice 21 days after receiving adenoassociated viral (AAV) vectors delivering both HBV and HDV genomes (HBV/HDV) at a dose of 5x10^10^ vg/mouse each. Representative images of hybridized liver sections were captured using the Vectra Polaris Automated Imaging System at various magnifications. In the last image a macrophage containing HDV RNA has been identify with a circle. (TIF) [file ppat.1011749.s002.TIF]

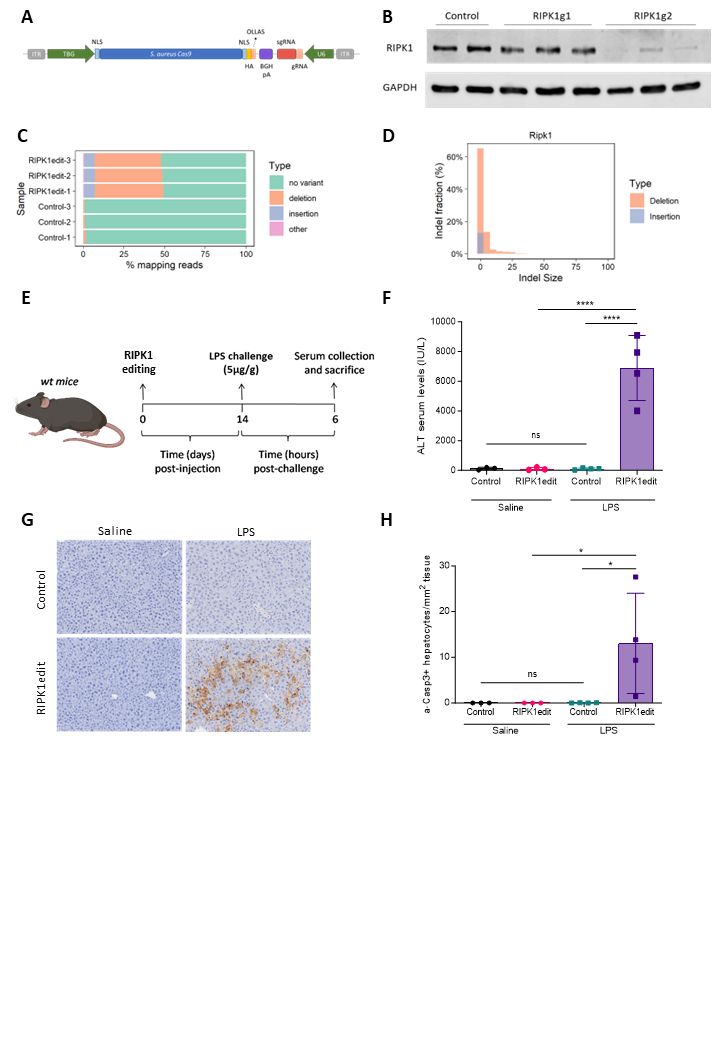

Supplement: S3 Fig — (A) Schematic representation of the recombinant AAV genome carrying the Staphylococcus aureus Cas9 protein flanked by two nuclear localization signals (NLS) and fused to the OLLAS tag under the control of a liver specific promoter TBG (human thyroxine binding globulin promoter) and the guide RNA (sgRNA + gRNA) under the control of U1 promoter. (B) C57BL/6 mice received 1011 vg of AAV-SaCas9-RIPK1g1 or AAV-SaCas9-RIPK1g2 and 30 hours later animals were sacrificed and RIPK1 expression was analyzed by western blot in liver extracts. (C) Bar chart of allele variant frequency (mean) analyzed in 3 control mice and 3 RIPK1edit mice. Green: no variant, orange: deletion, grey: insertions, pink: others. (D) Indel size distribution: orange for deletions and grey for insertions. (E) Schematic representation of the experimental procedure, 6/8-week-old C57BL/6 wt mice were iv injected with 1011 vg of AAV-SaCas9-RIPK1g2 (RIPK1edit) or an AAV expressing SaCas9 without guide (control) and 14 days later animals were challenged with LPS at a dose of 5 μg/gr and sacrificed 6 hours later. Liver damage was analyzed by (F) quantification of serum ALT levels (IU/L) and (G,H) quantification of a-Casp3+ hepatocytes/area after IHC analysis. Statistical analysis was performed by one-way ANOVA followed by Bonferroni multiple-comparison test. p <0.05 (*), p <0.0001 (****), ns = non-significant. (A) Created with BioRender. (TIF) [file ppat.1011749.s003.TIF]

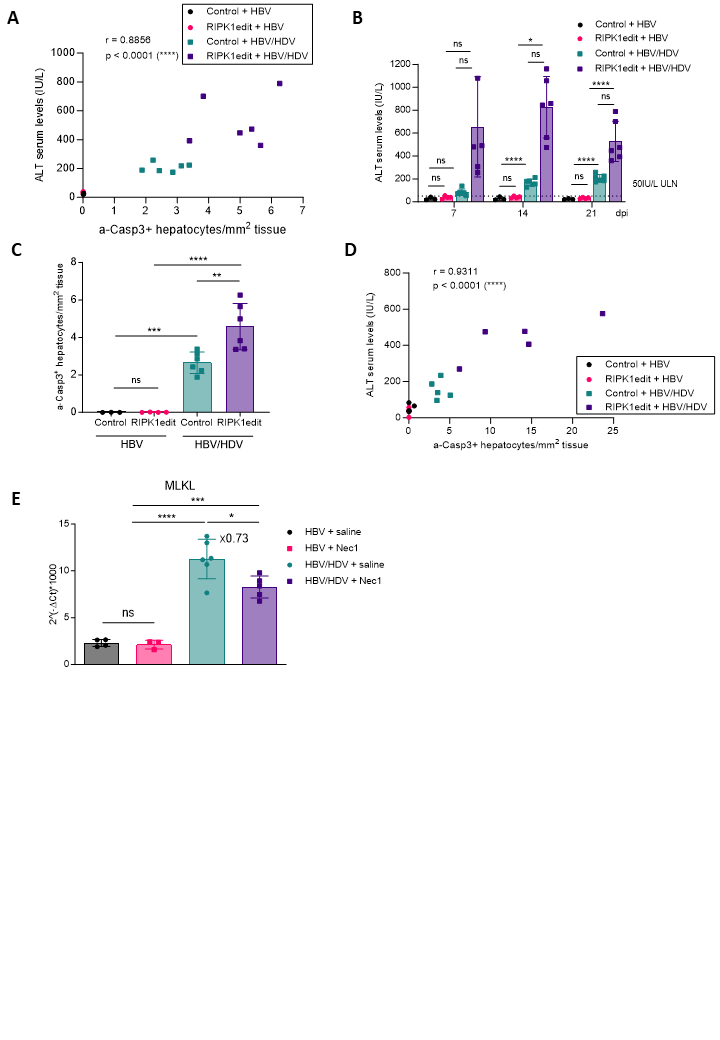

Supplement: S4 Fig — (A) Correlation between ALT levels and a-Casp3 positive hepatocytes in wt mice treated as described in Fig 2A. (B-D) C57BL/6 Rag1 KO mice were treated as described in Fig 2A and liver damage was analyzed by (B) quantification of serum ALT levels (U/L) and (C) quantification of a-Casp3+ hepatocytes/area after Immunohistochemistry (IHC) analysis. (D) Correlation between ALT levels and a-Casp3 positive hepatocytes. (E) MLKL expression levels was analyzed in the liver of mice treated daily with a dose of 2.5 mg/kg Nec1 or saline and that were previously injected with AAV-HBV (HBV) or AAV-HBV/HDV (HBV/HDV). Statistical analysis was performed by one-way ANOVA followed by Bonferroni multiple-comparison test. p <0.05 (*), p <0.01 (**), p <0.001 (***), p <0.0001 (****) and ns = non-significant. (TIF) [file ppat.1011749.s004.TIF]

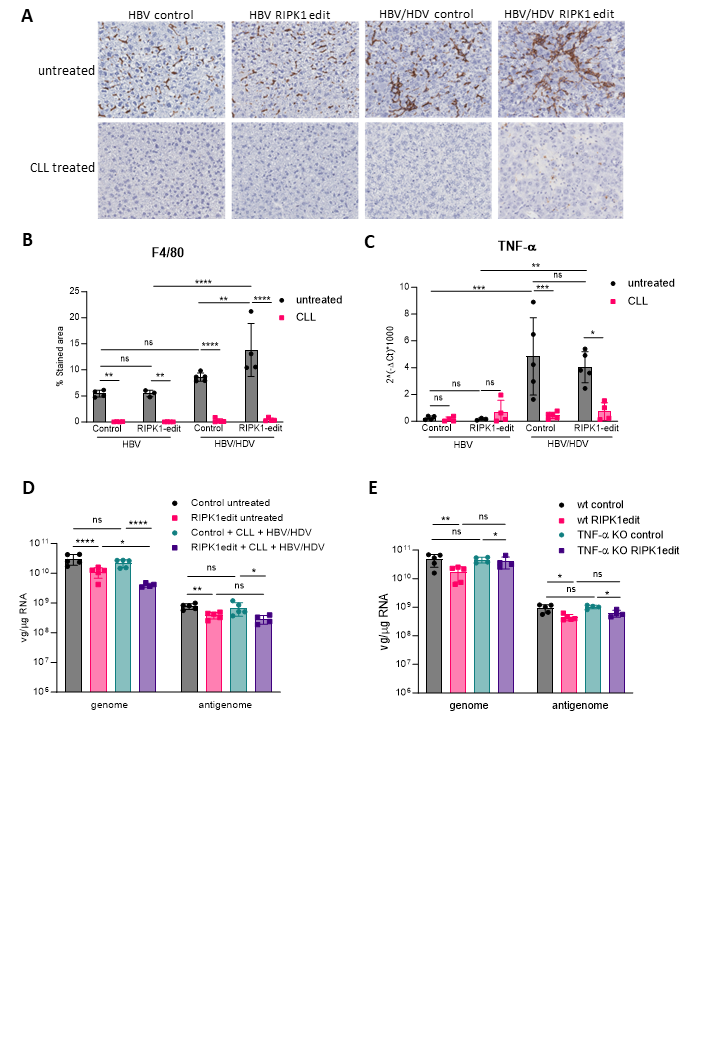

Supplement: S5 Fig — (A) IHC analysis against F4/80 was performed at 21 dpi in liver sections of mice receiving saline or clodronate loaded liposomes. Representative images from the different groups are shown. (B) quantitative analysis of F4/80 staining. (C) At sacrifice TNF-α expression was analyzed in the liver of mice by RT-PCR (D, E). The presence of HDV genomes and antigenomes in mouse liver samples was quantified by RT-PCR in CLL treated and untreated animals (D) and in TNF-α KO mice (E). Individual data points and mean values ± standard deviations are shown. Statistical differences were determined by two-way ANOVA. (TIF) [file ppat.1011749.s005.TIF]
