# Supplementary material for: Protective role of RIPK1 scaffolding against HDV-induced hepatocyte cell death and the significance of cytokines in mice
Source: PLoS Pathog. 2024 May 13;20(5):e1011749. doi: 10.1371/journal.ppat.1011749 (PMC11115361; doi:10.1371/journal.ppat.1011749)
Supplement: S2 Table — (DOCX) [file ppat.1011749.s007.docx]

S2 Table. Quantitative analysis of the ISH analysis performed in the liver of HBV/HDV mice using HDVg, HDVag and Albumin RNA probes and DAPI.
