# Supplementary material for: Protective role of RIPK1 scaffolding against HDV-induced hepatocyte cell death and the significance of cytokines in mice
Source: PLoS Pathog. 2024 May 13;20(5):e1011749. doi: 10.1371/journal.ppat.1011749 (PMC11115361; doi:10.1371/journal.ppat.1011749)
Supplement: S4 Table — (DOCX) [file ppat.1011749.s009.docx]

S4 Table. Quantitative analysis of the ISH analysis performed in the liver of HBV/HDV

mice using HDVg, Albumin and F4/80 RNA probes and DAPI.
