# Supplementary material for: Protective role of RIPK1 scaffolding against HDV-induced hepatocyte cell death and the significance of cytokines in mice
Source: PLoS Pathog. 2024 May 13;20(5):e1011749. doi: 10.1371/journal.ppat.1011749 (PMC11115361; doi:10.1371/journal.ppat.1011749)
Supplement: Attachment — Submitted filename: Camps et al Point by point answer to the Reviewers .pdf [file ppat.1011749.s011.pdf]

Point by point answer to the Reviewers

Reviewer #1: The manuscript, "Protective Role of RIPK1 Scaffolding against HDV-Induced Hepatocyte cell death and the Significance of Cytokines in Mice" by Camps et al. provides insights into hepatitis Delta-mediated liver injury in a mouse model. The authors use an AAV-based HBV/HDV infection model in mice to illustrate intra-hepatic pathological changes and the involvement of cytokines and their dependence on RIPK1-mediated signaling.

The present study provides valuable insights for the community. The HBV/HDV mouse model has been described before and has been here applied to study hepatocyte cell death. Overall, the manuscript is of high quality and the conclusion are based on the results.

Thank you very much to the reviewer for the positive feedback and for emphasizing the significance of our contribution to the field.

Specific points to improve the study are listed below:

Reviewer #1: Statistical assessment of image analysis:

It's unclear how many independent staining's or animals were used to quantify the number of HDV positive cells or the number of TNFa producing cells in Figure 1 (this apparently also includes the data analysis in other figures). The process is also not particularly well explained in the materials and methods section. The authors should thus provide a broad overview how their image analysis pipeline was set up and how statistical robustness was assessed, including the number of animals used for each experiment.

In the first experiment, we analyzed the livers of four individual C57BL/6 mice receiving AAV-HBV/HDV and stained them with TNFα RNA probe, HDV antigenome RNA probe, HDV genome RNA probe, and DAPI (Fig. 1A). We have included new images in Figure 1, and the results of the analysis have been detailed in S1 Table.

In the second experiment, we analyzed the livers of the same animals but stained them with anti-F40/8 antibody, TNFα RNA probe, HDV genome RNA probe, and DAPI. New images have been added to Figure 1C. The quantitative results of the analysis have been provided in S3 Table.

Two additional ISH analyses were performed. In the first one, conducted on liver samples from two of the animals, samples were hybridized with anti-albumin RNA probe to detect hepatocytes, and anti-HDV genome and antigenome RNA probes were used. The images have been included in S1 Fig, and the quantitative analysis is presented in S1 Table. In the second analysis, anti-albumin RNA probe and anti-HDV genome probe were combined with an anti-F4/80 RNA probe to detect macrophages. The images have been included in S2 Fig, and the quantitative analysis is presented in S4 Table.

We have provided details on the number of animals analyzed and the analysis procedure. In the tables, the total number of cells and all the data obtained from individual samples have been included. Additionally, we have improved the description in the materials and methods section and the image analysis pipeline.

RIPK1 knockdown

Although a RIPK1 knockdown phenotype is present, the authors should perform sequencing or IF staining to get an idea of the proportion of altered cells in their mouse model. Do the authors also think that overexpression of RIPK1 in this model could provide additional information about

its role in HDV pathogenesis? In addition, the authors should provide information on what NEC1 does, as this is not clear from the reading.

- We conducted next-generation sequencing analysis of the targeted edited sequence and observed that 50% of the alleles underwent editing (refer to S3C Fig). Moreover, the graph depicted in S3D Fig illustrates that the genetic modifications predominantly comprised small deletions and insertions, resulting in frameshift mutations, which account for the reduction in protein expression.
- Given that the promoter controlling Cas9 expression is hepatocyte-specific, non-parenchymal cells remain unedited. Consequently, the observation of a 50% editing rate indicates that a high percentage of hepatocytes have undergone editing.
- We attempted to analyze RIPK1 depletion using Immunohistochemistry analysis with two different antibodies, but unfortunately, we were unsuccessful. However, as depicted in S3B Fig, RIPK1 deletion was clearly evident through western blot analysis.
- In the discussion, we have incorporated the potential protective role of RIPK1 overexpression against HDV-induced damage.
- Additionally, we have provided an explanation in the introduction regarding Nec1 as a necroptosis inhibitor by inhibiting RIPK1 kinase activity.

Assessment of infectivity

In most of the animal experiments, the authors indicate pathological changes when mice with different genetic background or treatment are infected. However, in most of these experiments, viral load is not quantified. This should be done to evaluate the effects of viral replication in the context of treatment or genotype.

We have included the genome and antigenome copy numbers in the liver of all the animals included in the study and referenced this data in the results section.

Figure 1:

The IF images in Figure 1 were somewhat difficult to interpret because no cell borders or nuclear staining were present. Without these, it is not possible for the reader to quantify the number of cells present in the images. In addition, the total number of cells should also be provided to provide insight into the statistical power of the analysis.

Color code

Some of the colors may be difficult to interpret, especially pink and green when used in the same figure.

We have incorporated DAPI staining in all images to facilitate the identification of cells. Additionally, the use of the albumin probe enabled us to specifically identify hepatocytes (refer to S1 Fig and S2 Fig). Macrophages were distinctly identified through F4/80 IHC (refer to Fig 1C). To provide comprehensive quantitative analysis, we have included four tables as supplementary material containing data obtained from each mouse, including cell counts.

Figure 2

The Reference for figure 2E seems to be wrong:

“The efficacy of Nec-1 treatment was evidenced by a significant reduction of MLKL expression levels in the liver of mice (S2E Fig). As shown in Figure 2E, transaminase levels in HBV/HDV injected mice were not affected by Nec-1 treatment.”

This sentences have been modified and now reads:

“The efficacy of Nec-1 treatment was demonstrated by a notable reduction in MLKL expression levels in the liver of mice (refer to S2E Fig). However, as depicted in Figure 2H, Nec-1 treatment had no discernible effect on HDV-induced liver damage, as evidenced by the absence of differences in transaminase increase between the two groups.”

Since kinase inhibition with Nec1 does not replicate the knockout phenotype, the authors should either perform additional experiments to clarify how RIPK1 affects HDV infection or discuss this finding in more detail.

As the reviewer rightly indicates, RIPK1 kinase activity does not appear to be responsible for protecting hepatocytes from HDV-induced cell death. Therefore, it is likely that the protective effect is attributed to RIPK1's scaffolding function. In the majority of animals utilized in the study, RIPK1 downregulation resulted in a reduction of HDV antigen and viral genomes in the liver. This observation may suggest a direct interaction between RIPK1 and HDV, indicating that this protein is necessary for HDV replication. However, in IFNα/βR KO mice, RIPK1 knockdown had no discernible effect on the levels of HDV genomes. The primary distinction between these animals and the others (wild type, TNF-α KO mice, CLL depleted) lies in the exacerbation of liver damage. Hence, we hypothesize that the reduction of HDAg and HDV genomes in RIPK1-edited mice is a result of increased hepatocyte death.

Reviewer #2: In this manuscript Camps and colleagues describe their study of the protective role of RIPK1 scaffolding against HDV induced cell death. For this study they employed the AAV-HDV mouse model, which recapitulates many features of HBV/HDV infection in humans. While they have previously shown that HDV induced liver damage can be diminished upon TNF-a treatment they now show that macrophages are the major source of TNF-a during HDV infection. In the current study they show that RIPK1, a downstream molecule of the TNF receptor, partially rescues infected cells from apoptosis via its scaffolding function and not its kinase function. Knock-out or knock-down of RIPK1 resulted in increased HDV-induced liver damage, except in mice that were unresponsive to type-I IFN, indicating that RIPK1 prevented HDV liver damage caused by type-I interferon induced cell death. Moreover they showed that the protective effect of RIPK1 was not linked to TNFa, nor to macrophage activation as depletion of these cells did not impact the event of liver damage.

Overall this is an interesting in vivo study that sheds light on the molecular mechanisms of HDV-induced liver damage, and the protective role RIPK1 plays in this. The study seems well-executed but several aspects could be adjusted to improve the readability of the manuscript (see below).

Thank you very much to the reviewer for the kind words and valuable feedback.

Figure 1: the TNFa staining in panel A is not really clear. This picture should be increased in size to make the statement more strong. Panel B, picture 4: HDV antigenomes can be observed in what appears a macrophage. This would suggest that HDV can replicate in macrophages. Is this a rare event? Can this be attributed to macrophages that have engulfed a dying hepatocyte or an

apoptotic body originating from a hepatocyte? The text states that 5-10% of TNF-expressing macrophages contain HDV RNA. Is this HDV genome or antigenome? Based on figure 1B-picture 2 and 4 I cannot exclude that the HDV signal is not observed in hepatocytes. A co-staining for a hepatocyte marker could make this clear.

There are several hypothesis to explain the presence of HDV RNA in the macrophages. As indicate in the discussion "*Interestingly, we also found a non-negligible percentage of macrophages that were positive for HDV RNA and all of them expressed TNF-α. Since initial HDV RNA synthesis in our system is controlled by a liver-specific promoter, and mouse cells cannot be directly infected by circulating HDV infectious particles, this finding suggests that HDV RNA is transferred from hepatocytes to macrophages through an alternative mechanism and not by AAV-HDV-mediated transduction or HDV infection. It has recently been described that HDV can efficiently spread without envelopment through the proliferation of infected cells [29], so one possibility is that HDV-positive hepatocytes proliferate and spread HDV genomes to other cells, including macrophages. Another potential explanation is that macrophages uptake extracellular vesicles (EVs) produced by hepatocytes harboring HDV replication. The formation of EVs from HDV- and HBV-infected hepatocytes has been reported by other groups [30,31]. EVs can be taken up by monocytes, macrophages, and dendritic cells, inducing their activation [32].*"

Now we have included the following sentence: **However, given that the replication of HDV genomes is hepatocyte-independent [33], we cannot rule out the possibility that HDV replicates in macrophages, thereby directly activating the innate immune response in these cells.**

Furthermore, to differentiate between HDV+ macrophages and HDV+ hepatocytes, we conducted an additional ISH analysis on four HBV/HDV mice using an anti-albumin probe to detect hepatocytes, an anti-F4/80 probe to detect macrophages, and an anti-HDV genome probe along with DAPI. The images have been included in Figure S2 and the quantification of the data in Table S4, which indicates that 6-9% of liver macrophages exhibited an HDV RNA signal, consistent with the data presented in Figure 1D.

In addition, the authors should include a table with an overview of all cell types (hepatocytes, macrophages, HDV genome positive/negative, HDV antigenome +/-, TNF+/- , ... and their relative presence (%) in the total number of liver cells. Single cell analysis would provide a better view on this, but is perhaps too much/difficult to perform in the context of a revision of this manuscript.

We concur with the reviewer that conducting a single-cell transcriptomic analysis would be highly informative, and we are currently in the process of refining this approach. In the meantime, to address this point, we have conducted additional ISH analyses (refer to S1 Fig and S2 Fig) and performed automated quantitative analysis on all images. The data for each individual animal are presented in four supplementary tables.

Data related to Figure 2:

- was the effect of RIPK1 KO assessed in mice that only were 'infected' with HDV (without HBV)? Using the AAV model, one would expect that HDV can replicate without the presence of HBV. Also in this case, HDV particles would not be secreted and perhaps not or less be detected/taken up by macrophages?

The reviewer's assumption regarding HDV replication in the absence of HBV is accurate. In our initial publication outlining the model (Suarez-Amaran et al. J Hepatol. 2017), we indeed observed HDV replication and antigen expression in mice that received only AAV-HDV. Furthermore, it's crucial to highlight that in animals administered both AAV-HBV and AAV-HDV, HDV replication significantly impacts AAV and HBV DNA levels, leading to their near-undetectability by day 21.

Regrettably, we were unable to conduct the suggested experiment repetition due to ongoing renovations in our animal facility's safety level 3 room, which currently prevents us from performing experiments involving AAV-HBV or AAV-HDV in mice.

- panel E: please provide quantitative data, with for HDAg and intracellular genomes (with statistical analysis)

We have incorporated the quantitative analysis of HDAg-stained cells, along with the analysis of genomes and antigenomes in the liver of the animals, into Figure 2. Subsequently, we conducted statistical analysis and identified significant differences between the control and RIPK1-edit mice.

- same panel: is there a potential link between the presence of HDAg and RIPK1 protein (same cells or neighboring cells). A co-staining would elucidate this.

We attempted RIPK1 immunohistochemistry staining on liver samples, but unfortunately, we were unsuccessful. As a result, we are unable to provide a reliable answer to this question.

Experiments related to (lack of) involvement of RIPK1 kinase activity:

- Efficacy of New-1 treatment led to significant reduction of MLKL expression levels: please provide fold change.

We have included the fold change value in the graph.

- P10 2nd paragraph: reference to figure 2E should be figure 2F

This mistake has been corrected.

Figure 3:

- the overall order of the different panels should be more intuitive (from left to right, and from top to bottom; now a mix).

Figure has been modified to be more intuitive.

- ideally this figure starts with a schematic overview in panel A, similarly to figure 2

A schematic overview has been included in figure 3 (panel C)

p6, line 8: "HDV subtle " should be shuttle

The reviewer is right and it was corrected, thanks.
